# When Psychiatry Meets Cardiology: A Case Report on the Challenges of Diagnosing and Managing Cardiovascular Disease in Patients With Severe Mental Illness

**DOI:** 10.7759/cureus.79345

**Published:** 2025-02-20

**Authors:** Leyan Edhem, Mae Hands, Gedoni Eni, Shehnoor Kaur, Adnan Ahmed, Jhiamluka Solano

**Affiliations:** 1 Cardiology, Scunthorpe General Hospital, Scunthorpe, GBR; 2 Emergency Medicine, Scunthorpe General Hospital, Scunthorpe, GBR; 3 Internal Medicine, Scunthorpe General Hospital, Scunthorpe, GBR; 4 Internal Medicine, Sri Guru Ram Das Medical Institute of Medical Sciences and Research, Amritsar, IND; 5 Cardiology, Hull University Hospital (Castle Hill Hospital), Hull, GBR; 6 Education Committee, Academy of Medical Educators, Cardiff, GBR

**Keywords:** anticoagulation therapy, coronary artery aneurysm, diagnostic challenges, multidisciplinary management, severe mental illness

## Abstract

Coronary artery aneurysms (CAA) are rare vascular abnormalities defined as focal dilations exceeding 1.5 times the diameter of an adjacent normal segment. Serious complications of CAAs include thrombosis, rupture, and myocardial infarction. Despite the risk of severe complications, patients with CAAs are often asymptomatic and diagnosed incidentally during imaging for unrelated conditions. The presence of a severe mental illness (SMI) poses additional challenges in dealing with patients with cardiovascular disease. We present the case of a 68-year-old woman who was admitted to the emergency department with hemoptysis, hypoxia, tachycardia, and hypertension in addition to being agitated and uncooperative. A computed tomography pulmonary angiography revealed several concerning findings, most notably an aneurysmal structure located inferior to the right pulmonary artery. Several challenges were faced in further investigating and managing the CAA discovery. These resulted from concerns surrounding the patient’s ability to tolerate further investigations, the risk of poor compliance with medical management, and the limited capacity for invasive treatments. Following multi-disciplinary team discussions, conservative management was favored, and anticoagulation therapy was initiated. This case underscores the complexity of diagnosing and managing coronary artery aneurysms in patients with severe mental illness. The absence of standardized guidelines for CAAs further complicates management decisions, requiring a case-by-case approach. A holistic, patient-centered approach that integrates psychiatric, cardiovascular, and ethical considerations is essential in improving outcomes in this vulnerable population.

## Introduction

Coronary aneurysms are traditionally defined as a localized enlargement of a coronary artery segment whose diameter exceeds 1.5 times the size of the adjacent, non-dilated segments. According to the coronary artery aneurysm registry (CAAR), the prevalence worldwide is 0.35% [1]. In the context of mental health, cardiovascular disease has a mortality rate twice that of the general population due to the high burden of modifiable cardiovascular risk behaviors and conditions [2]. As a result, cardiovascular disease management becomes particularly challenging in this population, providing barriers to optimal therapies and complex patient needs. We present the case of a patient with an incidental finding of coronary aneurysm in the context of a severe mental health condition.

## Case presentation

We present the case of a 68-year-old female who was admitted to the emergency department (ED) following a transfer from an inpatient mental health unit due to hemoptysis with clots. Her medical history was significant for chronic obstructive pulmonary disease (COPD), eczema, and schizoaffective disorder, for which she was under Section 3 of the Mental Health Act due to a relapse of psychosis. She was unable to provide any history to the ED doctors. Upon review of her medical records, she had been experiencing intermittent hemoptysis for the past month, following a previous ED visit two months prior for pneumonia. On physical examination, she was agitated and uncooperative. Auscultation revealed reduced air entry in the right middle and lower lung zones, alongside a widespread wheeze. Her oxygen saturation on room air was 89%, her heart rate was 114 beats per minute, and her blood pressure was 168/54 mmHg. Initial investigations showed raised inflammatory markers and D-dimer (Table 1). The patient was commenced on empirical antibiotic therapy for a suspected chest infection.

**Table 1:** Initial investigations performed on admission

Investigation	Patient value	Normal range
Hemoglobin	110	115 - 165 g/L
White blood cells	16.4	4.0 - 11 x 10^9^/L
Platelets	601	150 - 400 x 10^9^/L
C-reactive protein	124	0 - 8 mg/L
D-dimer	1946	0 - 500 ng/mL
Urea	5	2.5 - 7.8 mmol/L
Creatinine	64	60 - 110 µmol/L
Anti-Jo-1 antibody	Negative	
Anti-La antibody	Negative	
Anti-SSA antibody	Positive	

**Figure 1:**
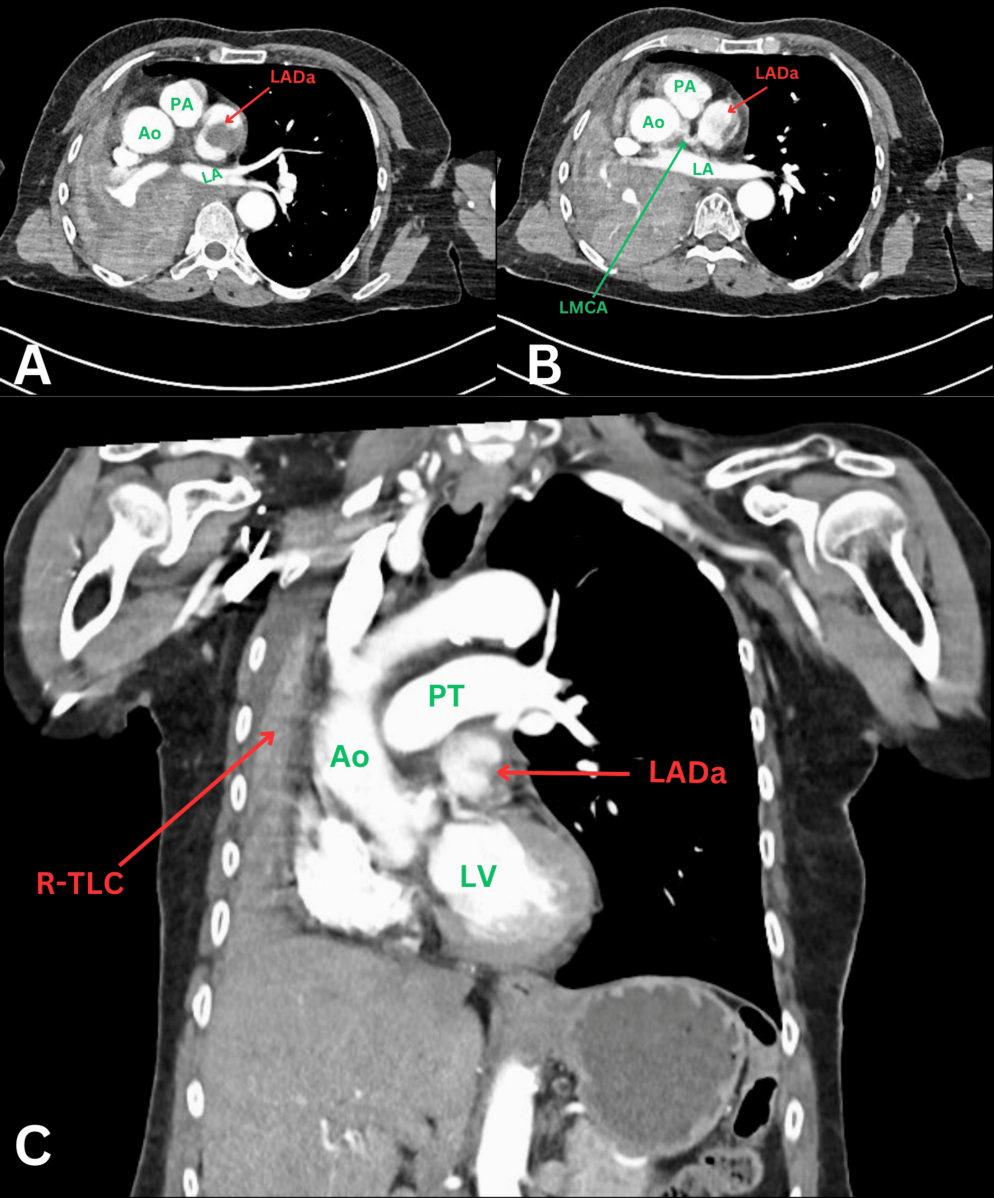
CT thorax with contrast showing coronary aneurysm and collapsed right lung A: axial CT thorax slice showing an aneurysmal structure with a central filling defect suspicious for a thrombus and contrast within the rest of the vessel lumen; B: axial CT slice showing the aneurysmal vessel as it branches off the left main coronary artery; C: coronal CT slice showing the relationship of the aneurysmal structure to the aorta and pulmonary trunk as well as a rightward mediastinal shift resulting from total right lung collapse. LADa: left anterior descending coronary artery aneurysm; PA: pulmonary artery; Ao: aorta; LA: left atrium; LMCA: left main coronary artery; PT: pulmonary trunk; LV: left ventricle; R-TLC: right total lung collapse

Computed tomography pulmonary angiography (CTPA) was performed during admission and revealed several concerning findings: a suspicious filling defect in the left atrial appendage, raising the possibility of a thrombus; complete collapse of the right lung, accompanied by a mediastinal shift; and complete occlusion of the right main bronchus with debris and a few enlarged mediastinal lymph nodes, likely reactive, were noted, though no masses were identified. Additionally, the CTPA highlighted the presence of an aneurysmal structure located inferior to the right pulmonary artery (RPA), measuring up to 4.2 cm x 2.9 cm with a central filling defect (Figure 1). Given the non-gated nature of the CT scan, which was affected by significant motion artifacts, the exact origin of this aneurysm could not be definitively determined. However, based on the anatomical location, it was suspected to be related to the left anterior descending artery (LAD), though the possibility of it representing a large right coronary artery aneurysm could not be ruled out. Due to the uncertainty surrounding its origin, further investigation was deemed necessary, and a coronary CT angiography (CTCA) was recommended for a more precise characterization of the aneurysm.

The Mental Health Liaison team reviewed the patient a few days later as part of the routine inpatient management for this patient. The team noted that, given the suspicion of a cardiac aneurysm, a Court of Protection order would be required if the risk of anesthesia and surgery was such that it could result in death. They advised that should conservative management be agreed upon, a best interests meeting should be held involving the patient’s brother and an independent mental capacity advocate (IMCA) before her return to the mental health unit.

Given the complexity of the situation, particularly concerning the patient’s challenges with compliance, the case was discussed at the coronary multi-disciplinary team (MDT) meeting. The primary team tried without success to get an echocardiogram or CTCA done. The MDT concluded that ongoing medical management was the preferred approach, as revascularisation was not indicated, given the absence of cardiovascular symptoms. They, however, accepted the option of a coronary CT angiogram (CTCA) under general anesthesia if requiring intervention by the cardiac surgery team. Following reluctance to initiate warfarin due to a lack of cooperation from the patient with blood tests, the team agreed to initiate anticoagulation therapy with edoxaban.

**Figure 2:**
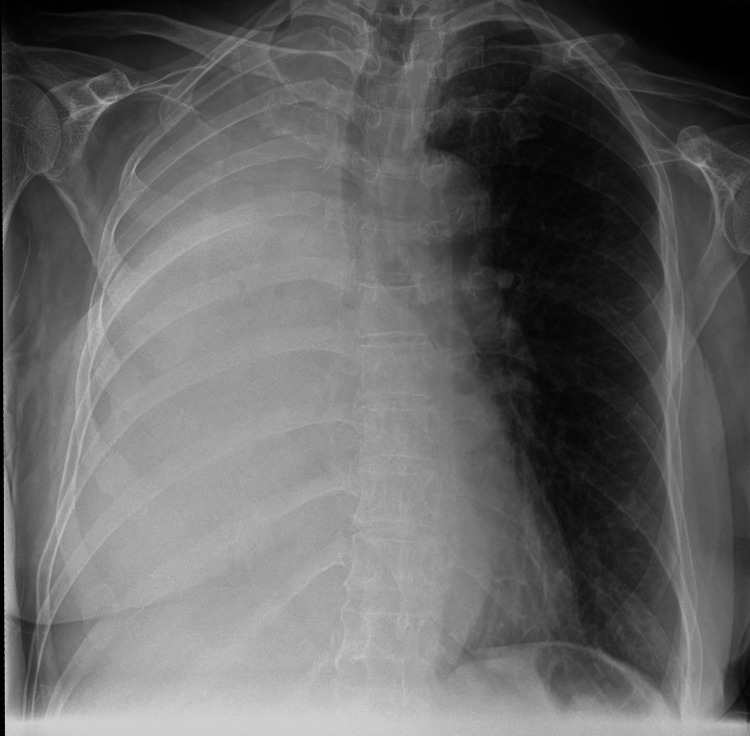
Anteroposterior chest X-ray showing complete whiteout of the right hemithorax due to right total lung collapse

With the help of the mental health nurses, a repeat chest X-ray was done (Figure 2), which revealed complete opacification of the right hemithorax, showing progressive changes since the previous imaging. As a result, the case, including the new radiological findings, was discussed at the lung MDT. The consensus was that no further interventions for her respiratory condition would be pursued, given her comorbidities, which would make her unable to tolerate any potential treatments. The wider MDT held a best interest meeting during which it was agreed that no further investigations, such as bronchoscopy or additional assessments, would be pursued, given the patient’s challenging mental health conditions and her inability to cope with further interventions. The respiratory team agreed with this approach, and the patient was subsequently discharged.

Four weeks later, she was readmitted with worsening dyspnea and initially treated for a chest infection, which was later presumed to be due to the progression of underlying undiagnosed malignancy. She received oral doxycycline for five days, with plans for discharge back to the mental health unit, but unfortunately, she passed away two days into the new admission.

## Discussion

The prevalence of CAA is estimated to range from 0.3% to 5%, with a higher occurrence in men and a tendency to affect the proximal coronary artery segments [3]. Atherosclerosis is the leading cause of CAA in adults, though other conditions such as Kawasaki disease, connective tissue disorders, and vasculitis have also been identified as contributing factors [4]. Although CAAs can lead to serious complications like thrombosis, rupture, or myocardial infarction, they are often asymptomatic and typically identified incidentally during imaging for unrelated conditions [5]. Chest pain (angina) is often the primary clinical manifestation in symptomatic patients [6]. This case of an incidental discovery of a large coronary aneurysm highlights the complexities of managing patients with CAAs and concurrent mental health illnesses.

Individuals with schizophrenia or schizoaffective disorder, as illustrated in this case, may exhibit anosognosia, a condition characterized by a lack of awareness or understanding, whether genuine or feigned, regarding the presence of illness or its symptoms [7]. Additionally, studies have shown that patients with severe mental illness (SMI) may have a reduced sensitivity to pain compared to those without an SMI [8,9]. These factors, independently or in combination, can contribute to delays or failures in seeking medical attention for concerning symptoms. In this case, potential symptoms associated with the coronary artery aneurysm may have gone unrecognized by the patient due to their schizoaffective disorder and recurrent psychosis. Moreover, the patient’s inability to provide a medical history upon presentation to the ED accentuates the challenges faced by individuals with an SMI in articulating their physical health concerns. 

As most coronary artery aneurysms are found incidentally, standardized guidelines on the gold standard for detecting them are not clearly outlined [1,3]. CT coronary angiography (CTCA), which was deliberated in our patient’s case, has gained popularity due to its minimally invasive nature compared to the other imaging modalities and its high sensitivity in the detection of CAAs [5]. Although CTCA has been described as the imaging of choice for long-term monitoring of CAAs, the increased risk of malignancy from repeated radiation exposure must be considered, especially in children.

There are currently no biomarkers for CAA. However, certain abnormalities warrant further investigation and, in the right context, may hint at a possibility of CAA. Notably, our patient tested positive for SS-A antibodies. A positive result for these antibodies may suggest a connective tissue disorder (CTD) such as systemic lupus erythematosus, Sjogren’s syndrome, and inflammatory myopathies. Although rare, a clear relationship between connective tissue disorders and coronary artery aneurysms is documented [10,11], suggesting our patient may have had a concomitant undiagnosed CTD, which led to the development of her CAA. The management of patients with coronary artery aneurysms (CAA) is challenging, primarily due to the limited understanding of its natural history and the scarcity of robust comparative data regarding existing treatment modalities [1,3].

Conservative management typically involves antiplatelet or anticoagulants to mitigate thrombotic risks associated with CAA. Interventional strategies, particularly percutaneous coronary intervention (PCI), have demonstrated promising outcomes in select patient populations. A retrospective study by Núñez-Gil et al. [1] highlighted a tendency towards intensive treatment regimens, including prolonged antiplatelet therapy, with favorable long-term outcomes observed in patients undergoing PCI, especially when drug-eluting stents were used. Similarly, Khubber et al. [12] reported significantly improved outcomes in patients treated with coronary artery bypass grafting (CABG) compared to those managed with medical therapy alone. However, their findings did not reveal a statistically significant difference in outcomes between CABG and PCI. It is important to note that invasive modalities are generally reserved for patients presenting with symptoms or complications directly attributable to CAA.

Despite these insights, the retrospective nature of these studies imposes inherent limitations on their ability to change practice. Moreover, while patients with CAA are predisposed to cardiovascular complications and major adverse cardiac events (MACE), the actual incidence of aneurysm-related complications appears relatively low. For instance, Núñez-Gil et al. [1] observed that few patients in their cohort experienced complications directly attributable to the aneurysm. Khubber et al. [12] reported a 30% incidence of major cardiac and cerebrovascular events, though most of these patients had concomitant coronary artery disease (CAD), potentially confounding the association.

Further data from a large international CAA registry [13] corroborated these findings, revealing that 22% of patients died from various causes and 37% experienced MACE. Interestingly, only 2% of the cohort suffered complications directly related to the aneurysm itself. These disparities highlight the complexities in treatment and prognostication alongside the lack of high-quality evidence to define standardized management protocols. Given the absence of definitive data and the conflicting results within the existing literature, individualization remains the mainstay approach in management. This relies heavily on multidisciplinary team (MDT) collaboration, with treatment decisions tailored to the patient’s clinical presentation, comorbidities, and available resources.

In our case, the patient was ‘asymptomatic,’ and the aneurysm was incidentally discovered while investigating for pulmonary pathology. After the MDT discussion, a conservative management strategy was adopted. Despite the presence of a cardiac thrombus, the team recommended a direct oral anticoagulant (DOAC) over warfarin, primarily due to concerns regarding the patient’s potential non-compliance with required monitoring. This decision reflects the nuanced, patient-centered approach in the presence of significant mental health problems and current gaps in evidence surrounding CAA management.

Challenges in managing coronary artery aneurysms in patients with severe mental illness

Pain Perception and Reporting Challenges

In this case, the patient’s caregivers did not report any chest pain, which influenced the decision to opt for conservative management. However, it was unclear whether the patient was truly asymptomatic or simply unable to communicate their symptoms. Research suggests that individuals with schizophrenia may have a diminished pain response due to impaired neurobiological pain transmission [14,15]. This reduced perception, combined with communication difficulties, can result in poor help-seeking behaviors. Given that chest pain is a key indicator of cardiovascular disease, failure to report symptoms can significantly impact the quality of care for this patient population.

Barriers to Cardiovascular Interventions

A major challenge in managing cardiovascular conditions in patients with severe mental illness is the disconnect between psychiatry and cardiology. Psychiatrists may have limited expertise in cardiovascular risk assessment, while cardiologists may not fully understand the complexities of treating patients with severe mental illness [2]. In this case, psychiatric consultation was required to assess decision-making capacity and determine the necessary documentation for investigations.

Proceeding with invasive procedures such as coronary angiography would have required general anesthesia, adding further complexity. The patient was deemed to lack capacity, and there were significant risks associated with the procedure, including death. Key concerns included the ability to consent, the appropriateness of invasive procedures, and adherence to post-procedural treatments such as antiplatelets or anticoagulation. As a result, following the MDT discussion, conservative management was preferred to avoid potential harm from intervention.

Reduced Access to Invasive Cardiovascular Treatments

Studies indicate that patients with severe mental illness are less likely to undergo invasive cardiovascular interventions. A meta-analysis by Mitchell and Lawrence found that individuals with mental illness had nearly a 50% lower likelihood of undergoing coronary procedures, with the disparity being particularly pronounced in schizophrenia [16]. This underutilization of interventions may be influenced by concerns about decision-making capacity, treatment adherence, and the risks associated with procedures, reinforcing the tendency toward conservative management in this patient group.

## Conclusions

This case highlights the complexity of detecting and managing CAAs. Given the lack of definitive guidelines, decisions regarding the management of CAAs should be made on a case-by-case basis, considering the patient’s comorbidities as well as psychosocial factors that may influence their ability to tolerate certain interventions. It emphasizes the significance of maintaining a holistic and patient-centered approach when considering investigative and management options, especially in patients with limited capacity for decision-making. Despite best efforts, the patient’s clinical deterioration and subsequent death serve as a reminder of the importance of early recognition, multidisciplinary team involvement, and individualized treatment planning.
